# Correlation between Serum Level of Monocyte Chemoattractant Protein-1 and Postoperative Recurrence of Spinal Tuberculosis in the Chinese Han Population

**DOI:** 10.1371/journal.pone.0125756

**Published:** 2015-05-11

**Authors:** Dan He, Xiaolu Zhang, Qile Gao, Rongfu Huang, Zhansheng Deng, Chaofeng Guo, Qiang Guo, Jia Huang, Hongqi Zhang

**Affiliations:** 1 Department of Neurology, The First Hospital of Changsha, Changsha, People’s Republic of China; 2 DepartmentofOrthopedics, The second affiliated Hospital, Fujian Medical University, Quanzhou, People’s Republic of China; 3 Department of Spine Surgery, Xiangya Spinal Surgery Center, Xiangya Hospital, Central South University, Changsha, People’s Republic of China; 4 Clinical Laboratory, The second affiliated Hospital, Fujian Medical University, Quanzhou, People’s Republic of China; University of Minnesota, UNITED STATES

## Abstract

**Objective:**

To correlate serum level of monocyte chemoattractant protein-1 (MCP-1) with postoperative recurrence of spinal tuberculosis in the Chinese Han population.

**Methods:**

Patients of Han nationality with newly diagnosed spinal tuberculosis were consecutively included in this study. At different time points postoperatively, serum level of MCP-1 was determined using an enzyme linked immunosorbent assay. Recurrence of spinal tuberculosis after surgery and during the follow-up period was recorded. The correlation between serum MCP-1 level and recurrence of spinal tuberculosis was analyzed.

**Results:**

A total of 169 patients with spinal tuberculosis were included in the study and followed up for an average of2.2±1.3 years (range, 1–5 years). Of these patients, 11 had postoperative recurrence of spinal tuberculosis. The patients’ serum level of MCP-1 increased significantly after postoperative recurrence of spinal tuberculosis. Once the symptoms of recurrence were cured, the serum level of MCP-1 decreased significantly and it did not differ from patients without disease recurrence.

**Conclusion:**

Postoperative recurrence of spinal tuberculosis is likely to increase the serum level of MCP-1.

## Introduction

Spinal tuberculosis is the most common form of skeletal system tuberculosis infection and it has a very high prevalence and morbidity in China [1]. As various anti-tuberculosis drugs poorly permeate foci and cold abscess in the vertebral body and do not always achieve effective therapeutic levels [2], it is difficult to treat spinal tuberculosis using drugs [3]. With surgery, the tuberculosis foci on which anti-tuberculosis medications are not effective, such as caseous necrotic tissue, abscess and dead bone, can be removed, but the *Mycobacterium tuberculosis* bacilli cannot be completely cleared. Thus, neither medication nor surgery is completely effective in removing spinal tuberculosis foci *in vivo*. Thus focus removal and even spinal tuberculosis recurrence are rarely avoided. Therefore, the immunity of the host after spinal tuberculosis surgery has a decisive influence on the patient’s clinical prognosis [4–6].

During the host’s *M*. *tuberculosis*-specific immune response, monocyte chemoattractant protein-1 (MCP-1) participates in the inhibition of *in vivo* dissemination of *M*. *tuberculosis*, regulating the secondary immune response.ThereforeMCP-1 is an important promoter and regulator of a host’s defense against tuberculosis infection. Our previous findings have shown that MCP-1 gene polymorphisms and serum MCP-1 level greatly influence the occurrence and development of spinal tuberculosis [7]. However, the role and effect of MCP-1 in a host’s defense against *M*. *tuberculosis* infection after surgery remain poorly understood. To the best of our knowledge, there have been no related reports in the literature. Therefore, we performed a postoperative follow-up observational study to further investigate the effect of MCP-1 serum level on the clinical outcome of spinal tuberculosis after surgery.

## Subjects and Methods

### Subjects

Patients of Han nationality with newly diagnosed spinal tuberculosis who received treatment between September 2009 and September 2013at the First Hospital of Changsha, Secondary Affiliated Hospital of Fujian Medical University and Xiangya Hospital of Central South University, both in China, were consecutively included in this study. The histopathological sections of all patients had features of spinal tuberculosis. Examination of the liquor puris demonstrated *M*. *tuberculosis* infection and positive acid-fast staining. Mycobacterium tuberculosis was isolated and cultivated by the Clinical Laboratory of Xiangya Hospital, China. Lowenstein Jensen Medium (L. J. Medium) was cultured. The colonies appeared on L.J. medium and they were confirmed by Ziehl-Neelsen staining. The isolated Mycobacterium tuberculosis in culture was genotyped by Centers for Disease Control and Prevention(CDC) of Hunan Province, China.

Patients who had any of the following conditions were rejected for this study: (1) drug-resistant tuberculosis; (2) diseases that influence the immune state, including infection, trauma and tumor; (3) diseases that influence MCP-1 expression, including coronary heart disease and psoriasis; (4) autoimmune diseases; (5) tuberculosis of other organs (or had a history of this disease); (6) hereditary disease; (7) spinal surgery; (8) not receiving the *Mycobacterium bovis* bacille Calmette–Guérin (BCG) vaccine.

The study protocol received approval from the Human Ethics Committee, Central South University, China. Written informed consent was obtained from each subject prior to inclusion in the study. The individual in this manuscript has given written informed consent (as outlined in PLOS consent form) to publish these case details.

### Methods

After regular anti-tuberculosis treatment, when blood sedimentation was normal or decreased, tuberculosis foci removal together with internal fixation and bone grafting was performed. At 1 and 2 weeks after surgery and during the follow-up period, 5 mL fasting blood was taken from the median cubital vein using a pro-coagulation tube in the morning, shaken slightly, and then centrifuged at 193.587 g (1000 rpm) at 4°C for 10minutes. For the patients who suffered from recurred disease, sample taking was necessary when they came back for treatment, at 1 and 2 weeks after being resolved and during the follow-up period. The supernatant was transferred to a 1.5-mL EppendorfSafe-Lockmicrocentrifuge tube and stored at −80°C. The MCP-1 concentration was determined using a human MCP-1 ELISA kit (Shanghai ExCellBioCo.,Ltd., China) according to manufacturer’s recommendations. The optical density was measured at 450nm using a microplate reader (Beckman Coulter, Inc., Brea, CA, USA). Recurrence of spinal tuberculosis after surgery and during the follow-up period was recorded in detail.

### Follow-up and observation

The patients without disease recurrence underwent examination with x-ray, CT, MRI and serum MCP-1 levels measured at 3, 6 and 12 months after surgery and then once per year. The patients with disease recurrence underwent examination with x-ray, CT, MRI and serum MCP-1 levels measured immediately after recurrence of spinal tuberculosis. When the disease recurrence was resolved, these patients were asked to undergo the above examinations once every 6 months.

### Statistical analysis

All data were statistically analyzed using SPSS 13.0 software(IBM Corp., Armonk, NY, USA). t-tests and one-way analysis of variance were used for comparison of MCP-1 concentration between groups. Data was normally distributed and expressed as the mean ± SD. A *P*-value of *P*< 0.05 was considered statistically significant.

## Results

### Patient characteristics

One hundred sixty-nine patients with spinal tuberculosis, consisting of 95 males and 74 females, aged 40.62±17.38 years (range, 2–78 years) were enrolled in the study. Through examination of x-ray, CT and MRI results, the tuberculosis lesions were located in the cervical vertebrae in 11 patients, in the T_1-9_ segments in 44 patients, in the T_10_-L_2_ segments in 47 patients, in the L_3-5_ segments in 51 patients, and in the L_5_-S_1_ in 16 patients.

### Patient follow-up and complications

**Fig 1:**
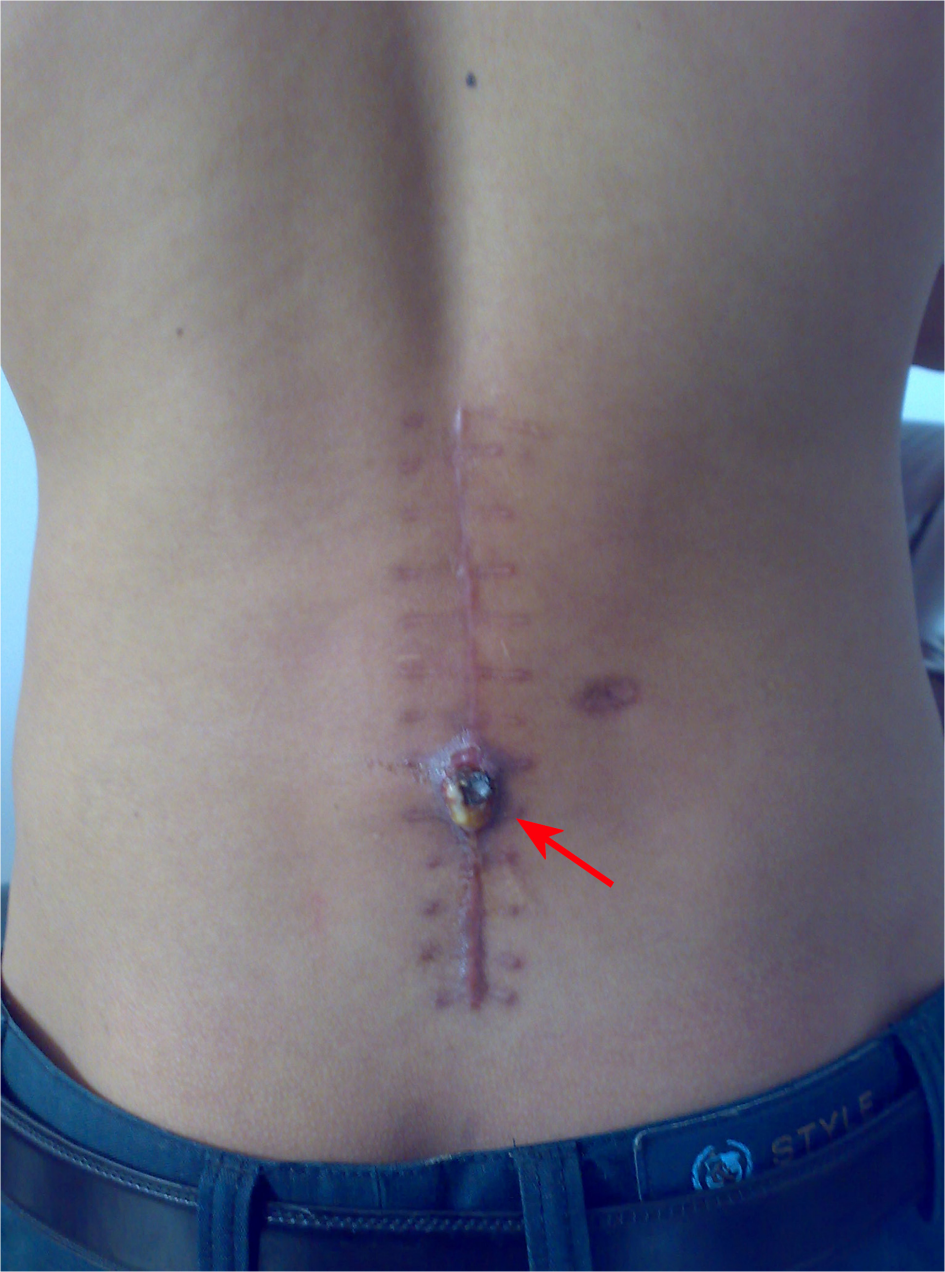
A sinus tract forms at the site of the incision after surgery.

**Fig 2:**
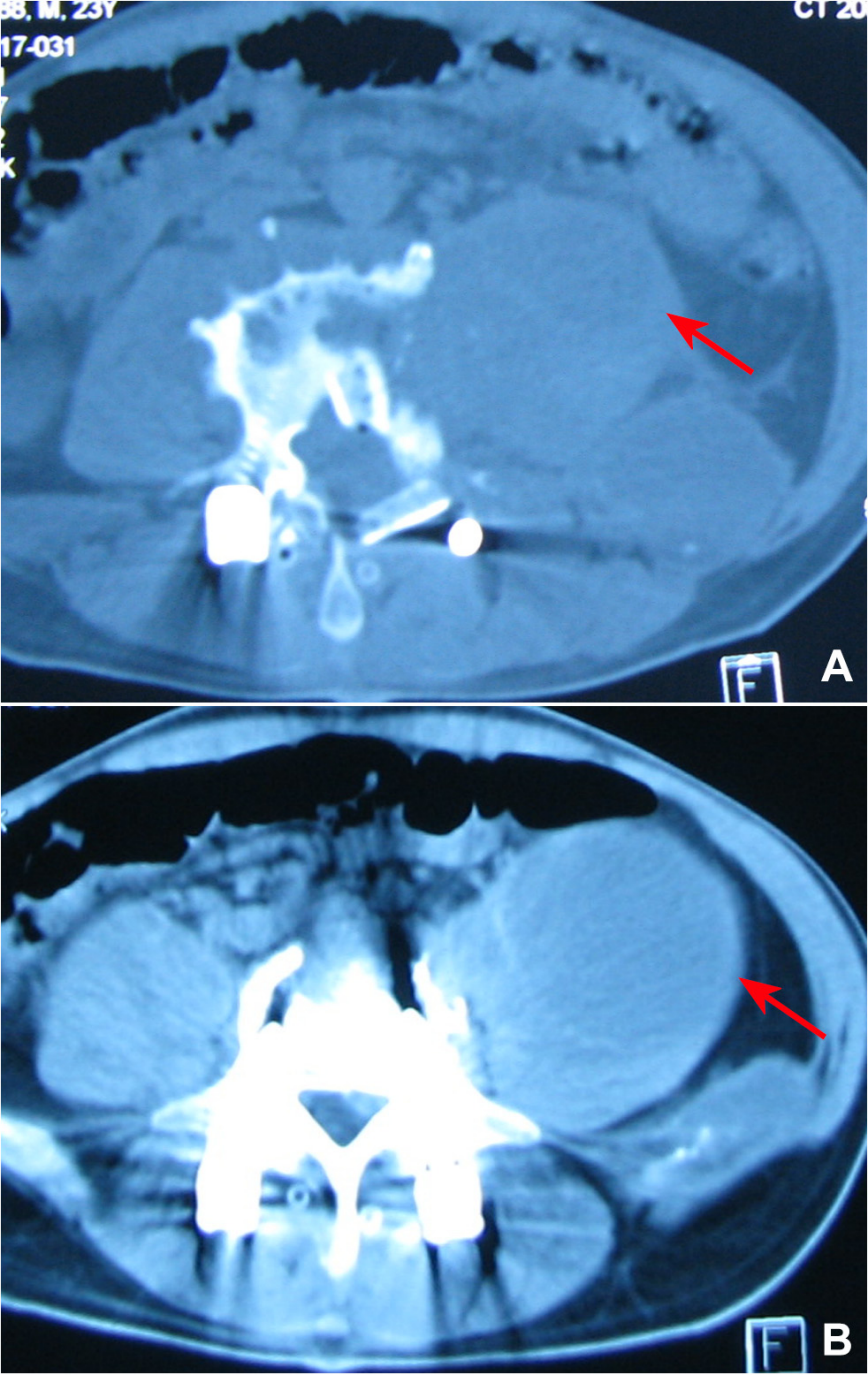
Iliopsoas abscess recurrence at 6 months after surgery. 2a: The iliopsoas abscess was almost gone immediately after surgery. 2b: iliopsoas abscess recurred at 6 months after surgery.

**Fig 3:**
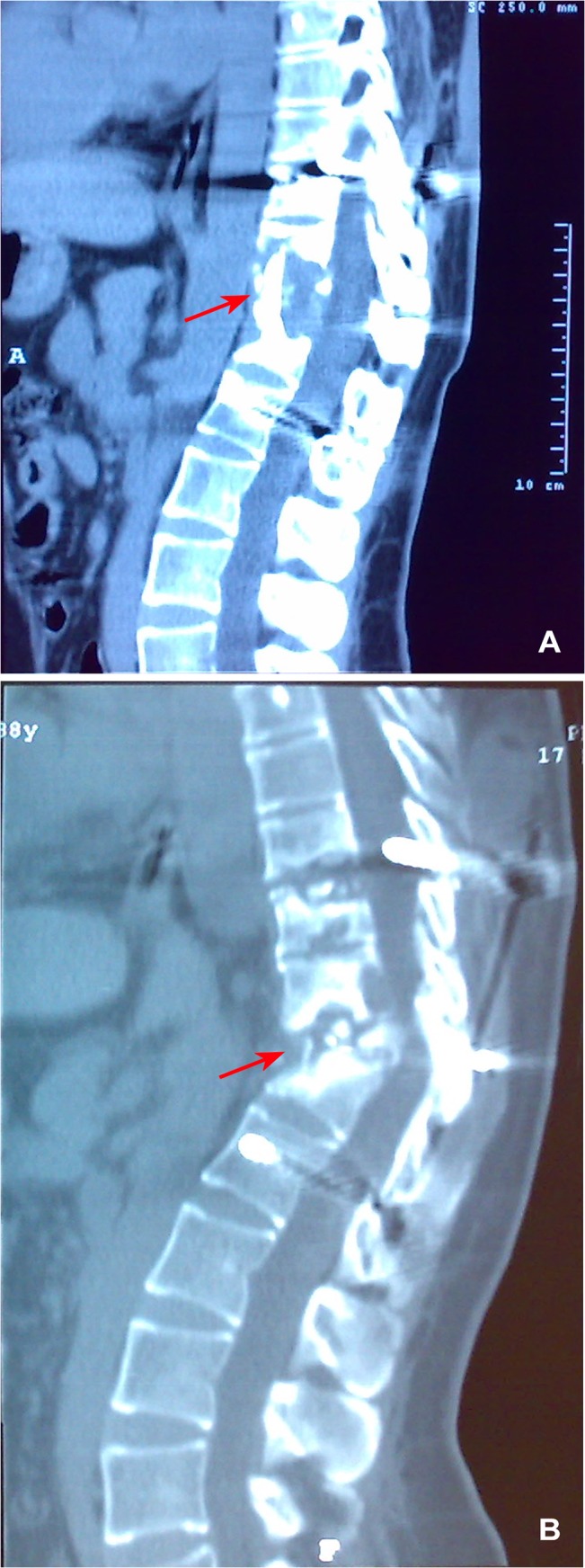
Bone grafts were absorbed and internal fixation failed. 3a: At 2 weeks after surgery, bone grafts were well positioned. 3b: at 1 year after surgery, bone grafts were absorbed and internal fixation failed.

All patients were followed for 2.2±1.3 years (range, 1–5 years). Eleven (6.51%) patients had postoperative recurrence of spinal tuberculosis, including fistula (n = 6; Fig 1), iliopsoas abscess (n = 3; Fig 2), and bone destruction (n = 2; Fig 3). Disease recurrence in these patients was resolved after active treatment and had not reoccurred at the last follow-up.

### Comparison of serum levels of MCP-1

#### Serum level of MCP-1 in patients with recurrence of spinal tuberculosis

Serum level of MCP-1 was significantly increased after recurrence of spinal tuberculosis when compared with the previous follow-up (t = 2.463, *P* = 0.023). When the symptoms of recurrence were resolved, serum level of MCP-1 was significantly decreased at the last follow-up compared with both the period of disease recurrence (t = -5.614, *P*<0.01) and prior to the onset of recurrence (t = -5.79, *P*<0.01; Table 1).

**Table 1:** Changes in serum MCP-1 level in patients with spinal tuberculosis

Group	Time point	Serum level of MCP-1 (ng/L)
Without disease recurrence (n = 158)	Last follow-up (ranged from 1 to 5 years)	51.04±10.63
With disease recurrence (n = 11)	Last follow-up before recurrence of tuberculosis (ranged from 0.5 to 18 months)	109.5±28.33
	During the period of disease recurrence (ranged from 1 to 24 months)	157.86±58.65
	Last follow-up after recurrence was resolved (ranged from 1 to 5 years)	57.25±9.66

MCP-1: Monocyte chemoattractant protein-1.

#### Serum level of MCP-1 in patients with and without disease recurrence

Serum level of MCP-1 was significantly increased in patients who would develop recurrent disease compared with patients without recurrence at follow-up (t = 6.034, *P*<0.01). When the recurrent disease was resolved, there was no significant difference in serum MCP-1 level at the last follow-up between patients with and without disease recurrence (t = 1.88, *P* = 0.062; Table 1).

## Discussion

In patients with spinal tuberculosis, the originally closed vomica will be opened after surgery, which unavoidably results in *M*. *tuberculosis* entering into the circulation and triggering a systemic immune response. In addition, after removal of liquor puris and dead bone, the balance between the host's resistance and bacterial virulence will be destroyed. Thus, the host will attack *M*. *tuberculosis*, and a large number of inflammatory cells will begin to migrate from the systemic circulation to the focus cavity and phagocytose *M*. *tuberculosis*. After using anti-tuberculosis drugs, the host is able to remove any residual *M*. *tuberculosis*, resulting in clinical resolution of infection. Therefore, patient immunity is a key factor in reducing postoperative recurrence and influencing the prognosis [8,9].

MCP-1 (chemokine (C-C motif) ligand 2, CCL2) is a chemokine involved in Th1 and Th2 immunity and regulates immune cell function [10]. MCP-1 influences the occurrence and development of spinal tuberculosis through various regulatory pathways. Specifically, it can both inhibit the occurrence of spinal tuberculosis and also promote the development of this disease, with the final regulatory result depending on both the time window of MCP-1 expression and the serum level of MCP-1 [11, 12]. There is evidence that the serum level of MCP-1 increases to varying degrees in patients with tuberculosis [13,14]. Hasan et al [12] confirmed that serum MCP-1 level gradually increases with the aggravation of disease severity in patients with tuberculosis. Furthermore, Su et al. [15] found that the serum level of MCP-1 was more significantly elevated in late tuberculosis responders than in early tuberculosis responders. However it was normal in all patients after resolution of their tuberculosis infections. Our results also showed that the serum level of MCP-1 was significantly increased after the recurrence of spinal tuberculosis, and decreased after the recurrence was cured. Similarly, there were no significant differences in serum MCP-1 level at the last follow-up between patients with and without disease recurrence.

Shigenaga et al[16] showed that in reinfected dermal Calmette-Guérin lesions, MCP-1 levels peaked at 3 hours and were then down-regulated. Sustained overexpression of MCP-1 can initiate a series of adverse events. High levels of MCP-1 will limit monocyte responses and induce transforming growth factor-βexpression, selectively inhibiting Th1 immune responses bydecreasinginterleukin-12 (IL-12) and interferon-γ expression, and also influencing the formation of granuloma[17]. Ganachari et al[18] showed that MCP-1 can increase the expression of matrix metalloproteinase-1 in *M*. *tuberculosis* stimulated THP-1 cells. Matrix metalloproteinase-1, through protease-activated receptor-1 activation, triggers a hyperinflammatory response to *M*. *tuberculosis* infection. Flores-Villanueva et al [19] also reported that high levels of MCP-1 can inhibit the production of IL-12p40, which acts as a chemoattractant for macrophages and promotes the migration of bacterially stimulated dendritic cells [20].

Overexpression of MCP-1 can saturate target cells, causing them to lose sensitivity to the ligand, leading to changes in the MCP-1 concentration gradient and finally inhibiting the chemotactic response of monocytes [21]. MCP-1 overexpression can also up-regulate IL-4 expression, inducing the transformation of Th0 cells into Th2 cells, up-regulating the Th2 immune response and leading to further inhibition of the Th1 immune response *in vivo* [22]. These signaling pathways are likely to increase the risk of *in vivo* dissemination of *M*. *tuberculosis* and even lead to the recurrence of tuberculosis infection.

Yang’s study [23] showed the same symptoms and a similar rate of disease recurrence. However, abscess accounted for 74.4% of all recurrent manifestations in that study. In our study the rate of abscess is only 27.3% in patients with recurrent disease. This is owing to a longer duration of preoperative and postoperative chemotherapy [24,25].Additionally, current studies on serum MCP-1 levels are performed mainly in patients with pulmonary tuberculosis. We report data on the correlation between serum MCP-1 levels and postoperative recurrence of spinal tuberculosis in the Chinese Han population. Related studies are underway. We believe that with the prolongation of the follow-up period, an increase in sample size and the development of animal experiments, the mechanism by which serum MCP-1 level influences postoperative complications in spinal tuberculosis will be further clarified. This will provide a novel strategy for preventing and treating postoperative complications in spinal tuberculosis.

## Conclusions

In conclusion, our results suggest that the postoperative recurrence of spinal tuberculosis is likely to increase the serum level of MCP-1 in the Chinese Han population.

## Supporting Information

S1 Table Individual data points. (XLSX)

## References

[1] Garg RK, Somvanshi DS. Spinal tuberculosis: a review. J Spinal Cord Med. 2011; 34(5):440–454. 10.1179/2045772311Y.0000000023 22118251 PMC3184481

[2] Liu P, Zhu Q, Jiang J. Distribution of three antituberculous drugs and their metabolites in different parts of pathological vertebrae with spinal tuberculosis. Spine (Phila Pa 1976). 2011; 36(20):E1290–1295. 10.1097/BRS.0b013e31820beae3 21311403

[3] Valsalan R, Purushothaman R, Raveendran M, Zacharia B, Surendran S. Efficacy of directly observed treatment short-course intermittent regimen in spinal tuberculosis. Indian J Orthop. 2012 46(2):138–144. 10.4103/0019-5413.93673 22448050 PMC3308653

[4] Huynh KK, Joshi SA, Brown EJ. A delicate dance: host response to mycobacteria. CurrOpinImmunol. 2011; 23(4):464–472. 10.1016/j.coi.2011.06.002 21726990

[5] Natarajan K, Kundu M, Sharma P, Basu J. Innate immune responses to M. tuberculosis infection. Tuberculosis (Edinb). 2011; 91(5):427–431. 10.1016/j.tube.2011.04.003 21550855

[6] Behar SM, Martin CJ, Nunes-Alves C, Divangahi M, Remold HG. Lipids, apoptosis, and cross-presentation: links in the chain of host defense against Mycobacterium tuberculosis. Microbes Infect. 2011; 13(8–9):749–756. 10.1016/j.micinf.2011.07.006 21458584 PMC3130819

[7] Guo C, Zhang H, Gao Q, He D, Tang M, Liu S, et al Monocyte chemoattractant protein-1 in spinal tuberculosis: -362G/C genetic variant and protein levels in Chinese patients. DiagnMicrobiol Infect Dis. 2014; 78(1):49–52. 10.1016/j.diagmicrobio.2013.07.024 24183600

[8] Kleinnijenhuis J, Oosting M, Joosten LA, Netea MG, Van Crevel R. Innate immune recognition of Mycobacterium tuberculosis. Clin Dev Immunol. 2011; 2011: 405310: 10.1155/2011/405310 21603213 PMC3095423

[9] Li L, Zhang Z, Luo F, Xu J, Cheng P, Wu Z, et al Management of drug-resistant spinal tuberculosis with a combination of surgery and individualised chemotherapy: a retrospective analysis of thirty-five patients. IntOrthop. 2012; 36(2):277–283. 10.1007/s00264-011-1398-0 22065055 PMC3282866

[10] Siveke JT, Hamann A, T helper 1 and T helper 2 cells respond differentially to chemokines. J Immunol. 1998; 160(2):550–554. 9551886

[11] Deshmane SL, Kremlev S, Amini S, Sawaya BE. Monocyte chemoattractant protein-1 (MCP-1): an overview. J Interferon Cytokine Res. 2009; 29(6):313–326. 10.1089/jir.2008.0027 19441883 PMC2755091

[12] Hasan Z, Cliff JM, Dockrell HM, Jamil B, Irfan M, Ashraf M, et al CCL2 responses to Mycobacterium tuberculosis are associated with disease severity in tuberculosis. PLOS One. 2009; 4(12):e8459 10.1371/journal.pone.0008459 20041183 PMC2793516

[13] Hussain R, Ansari A, Talat N, Hasan Z, Dawood G. CCL2/MCP-I genotype-phenotype relationship in latent tuberculosis infection. PLOS One. 2011; 6(10):e25803 10.1371/journal.pone.0025803 21991356 PMC3186769

[14] Feng WX, Mokrousov I, Wang BB, Nelson H, Jiao WW, Wang J, et al Tag SNP polymorphism of CCL2 and its role in clinical tuberculosis in Han Chinese pediatric population. PLOS One. 2011; 6(2):e14652 10.1371/journal.pone.0014652 21556333 PMC3084193

[15] Su WL, Perng WC, Huang CH, Yang CY, Wu CP, Chen JH, Association of reduced tumor necrosis factor alpha, gamma interferon, and interleukin-1beta (IL-1beta) but increased IL-10 expression with improved chest radiography in patients with pulmonary tuberculosis. Clin Vaccine Immunol. 2010; 17(2):223–231. 10.1128/CVI.00381-09 20007364 PMC2815523

[16] Shigenaga T, Dannenberg AM, Lowrie DB, Said W, Urist MJ, Abbey H, et al Immune responses in tuberculosis: antibodies and CD4-CD8 lymphocytes with vascular adhesion molecules and cytokines (chemokines) cause a rapid antigen-specific cell infiltration at sites of bacillus Calmette-Guérin reinfection. Immunology. 2001; 102(4):466–479. 11328381 10.1046/j.1365-2567.2001.01195.x PMC1783201

[17] Shukla S, Singh S, Puri V, Verma D, Jain L. Bilateral symmetrical facial swelling owing to tuberculous gummas. Ann Trop Paediatr. 2011; 31(4):363–365. 10.1179/1465328111Y.0000000034 22041472

[18] Ganachari M, Guio H, Zhao N, Flores-Villanueva PO. Host gene-encoded severe lung TB: from genes to the potential pathways. Genes Immun. 2012; 13(8):605–620. 10.1038/gene.2012.39 22992722 PMC3518758

[19] Flores-Villanueva PO, Ruiz-Morales JA, Song CH, Flores LM, Jo EK, Montaño M, et al A functional promoter polymorphism in monocyte chemoattractant protein-1 is associated with increased susceptibility to pulmonary tuberculosis. J Exp Med. 2005; 202(12):1649–1658. 16352737 10.1084/jem.20050126 PMC2212957

[20] Cooper AM, Khader SA. IL-12p40: an inherently agonistic cytokine. Trends Immunol. 2007; 28(1):33–38. 17126601 10.1016/j.it.2006.11.002

[21] Charo IF, Ransohoff RM. The many roles of chemokines and chemokine receptors in inflammation. N Engl J Med. 2006; 354(6):610–621. 16467548 10.1056/NEJMra052723

[22] Mendez A, Hernandez-Pando R, Contreras S, Aguilar D, Rook GA. CCL2, CCL18 and sIL-4R in renal, meningeal and pulmonary TB; a 2 year study of patients and contacts. Tuberculosis (Edinb), 2011; 91(2):140–145. 10.1016/j.tube.2010.12.008 21251883

[23] Yang L, Liu Z. Analysis and therapeutic schedule of the postoperative recurrence of bone tuberculosis. J OrthopSurg Res. 2013; 8:47 10.1186/1749-799X-8-47 24341624 PMC3878563

[24] Zhang HQ, Li JS, Zhao SS, Shao YX, Liu SH, Gao Q, et al Surgical management for thoracic spinal tuberculosis in the elderly: posterior only versus combined posterior and anterior approaches. Arch Orthop Trauma Surg. 2012; 132(12):1717–1723. 10.1007/s00402-012-1618-0 23053192

[25] Zhang H, Sheng B, Tang M, Guo C, Liu S, Huang S, et al One-stage surgical treatment for upper thoracic spinal tuberculosis by internal fixation, debridement, and combined interbody and posterior fusion via posterior-only approach. Eur Spine J. 2013; 22(3):616–623. 10.1007/s00586-012-2470-1 22903198 PMC3585644

